# A study of the medicinal plants used by the Marakwet Community in Kenya

**DOI:** 10.1186/1746-4269-10-24

**Published:** 2014-02-20

**Authors:** Wilson Kipkore, Bernard Wanjohi, Hillary Rono, Gabriel Kigen

**Affiliations:** 1 Department of Forestry, University of Eldoret, P.O. Box 1125, Eldoret, Kenya; 2 Department of Wildlife Management, University of Eldoret, P.O. Box 1125, Eldoret, Kenya; 3 Ophthalmologist, Kitale Hospital and North Rift Zonal eye surgeon; Ministry of Health, P.O. Box 98, Kitale, Kenya; 4 Department of Pharmacology and Toxicology, Moi University School of Medicine, P.O. Box 4606, Eldoret, Kenya

**Keywords:** Marakwet, Herbal medicine, Ethnobotanical, Plants, Documentation, Research

## Abstract

**Background:**

The medicinal plants used by herbalists in Kenya have not been well documented, despite their widespread use. The threat of complete disappearance of the knowledge on herbal medicine from factors such as deforestation, lack of proper regulation, overexploitation and sociocultural issues warrants an urgent need to document the information. The purpose of the study was to document information on medicinal plants used by herbalists in Marakwet District towards the utilization of indigenous ethnobotanical knowledge for the advancement of biomedical research and development.

**Methods:**

Semi- structured oral interviews were conducted with 112 practicing herbalists. The types of plants used were identified and the conditions treated recorded.

**Results:**

Herbal practice is still common in the district, and 111 plants were identified to have medicinal or related uses. Different herbal preparations including fruits and healing vegetables are employed in the treatment of various medical conditions. Veterinary uses and pesticides were also recorded.

**Conclusion:**

The study provides comprehensive ethnobotanical information about herbal medicine and healing methods among the Marakwet community. The identification of the active ingredients of the plants used by the herbalists may provide some useful leads for the development of new drugs.

## Background

Medicinal plants have been used by humans from time immemorial. Many drugs have plant origin, and several plants are currently undergoing investigation to ascertain their therapeutic efficacies [1-3]. Traditional herbal medicine is still an important component of healthcare in sub-Saharan Africa. This is largely due to poverty, inadequacy of health services and shortage of health workers. Even when the facilities exist, there is rampant shortage of drugs and equipment. The World Health Organization (WHO) estimates that up to 80% of the population in some developing countries use traditional medicine [4,5].

Like many other developing countries, the use of traditional herbal medicine is still widespread in Kenya, especially in the rural areas [4]. In some instances, herbal remedies are combined with conventional medicine especially when the subjects feel that the prescription drugs are not effective [6]. Despite this, most of the ethnobotanical information on herbal medicine and healing methods largely remain undocumented. This is compounded by the fact that in most Kenyan communities, the information is passed on orally, and only to very close relatives who might not necessarily be interested in practicing the art [7]. Lack of proper regulation has also led to the emergence of quack herbalists. The wanton destruction of forests and use of modern medicine have also contributed to the risk of the information disappearing completely over time [8]. There is therefore urgent need to document the knowledge on herbal information used by different communities in Kenya [7].

The Marakwet are a sub-tribe of the larger Kalenjin community residing in Rift Valley region. The other members of the Kalenjin ethnic group include Kipsigis, Nandi, Keiyo, Tugen, Pokot, Terik and Sabaot. They are Highland Nilotes, and are culturally related to the Maasai and Samburu. Apart from the Sabaot, all Kalejins live in the Great Rift Valley. The Sabaot live around Mt Elgon and are therefore citizens of both Kenya and Uganda. They are excellent athletes, and provide the vast majority of the Kenyan and of late Ugandan athletes with international honours in long distance races [9-11]. The Marakwet are mainly semi-pastoral just like the other Kalenjins [12]; Among the Kalenjin community, Marakwet District is one of the regions with a considerable number of authentic practicing herbalists. This is unlike many other regions of Kenya where there has been rapid decline in the use of herbal medicine. The local herbalists complement conventional local doctors in the treatment of common diseases within the district.

The perception of disease among the Marakwet like many other African Communities, is a complex matter. Disease or illness is thought to be associated with pollutants, misfortune, curse, “*Bon*” (witchcraft or sorcerers), or people with “bad eyes;” “*bich cho tinye konyen*”. In most cases, these people (both men and women) are not even aware that they have “bad eyes”. Usually they discover with horror that they can cause harm without their intention by merely looking at an object or person [13]. Each disease is therefore categorized according to the source, and the treatment differs. The practice of sorcery is not very common among the Marakwet. Treatment is by witchdoctors who use several methods including sacrificial animals. Preventive charms are used to protect against witches and people with “bad eyes”. Disease causing pollutants are treated by herbalists [13]. Pollutants, which are thought to emanate from such conditions as changes in weather; poisoning, insect bites, food and sex are treated by use of herbs. Currently, traditional herbal medicine is still viewed as complimentary to modern medicine. Amongst the Marakwet and their Keiyo counterparts, mental illness, infertility in women, chronic diseases such as diabetes and fractured skulls are reserved for traditional healers, whereas diseases such respiratory diseases including, pneumonia, fever and accidents are treated by modern medicine [13,14].

The use of Ethnobotanical medicines has been part and parcel of the health system of the Marakwet community from time immemorial. It has had profound socio-economic, cultural and educational values, which in turn has provided opportunities for enhancing their livelihoods within the Greater Rift Valley [13]. Historically, the Marakwet had an integrated health system that comprised of both herbal medicine and surgery. The herbalists treated common ailments, whereas the surgeons performed simple, and sometimes complicated surgical procedures such as craniotomy and tonsillectomy. The only other Kenyan communities on record to have practiced this form of specialized surgery are the Kisii, Meru and Kuria [14,15].

However most of the herbal preparations have not been recorded. Dr Lindsay, a missionary who worked at Kapsowar Mission Hospital for over 20 years published a book on Medicinal plants used by the Marakwet people which was however not exhaustive [16]. This is largely due to the fact that the practice is in most cases a closely guarded family secret. For instance, herbalists from the same locality may use similar plants to treat different conditions. Also, being a foreigner and missionary, Dr Lindsay faced several challenges in convincing the herbalists to reveal the herbs despite his long stay in the region. This was in part attributed to the negative approach by colonialists to traditional medicine who associated traditional healers with witchcraft or “black magic” and superstition [17]. Most of the herbalists with whom he interacted with are those who had converted to Christianity, mainly residing in the highlands around Kapsowar. Some few Marakwet herbal medicinal plants have also been reported by Kokwaro [18]. There are also published reports about traditional herbal medicine which have been carried out among the other Kalenjin tribes, notably the Nandi and Sabaot [19-21].

## Materials and methods

### Study area

**Figure 1:**
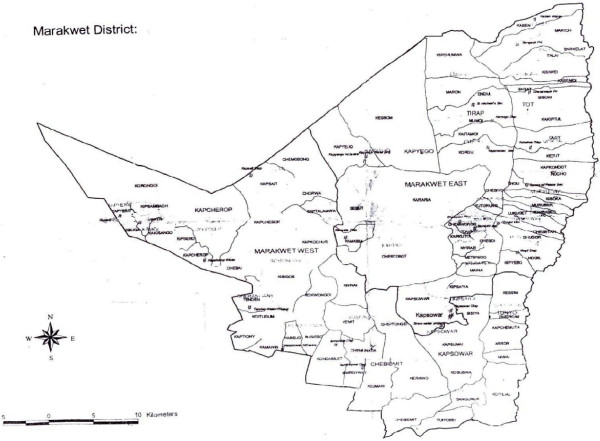
Map of Marakwet District.

**Figure 2:**
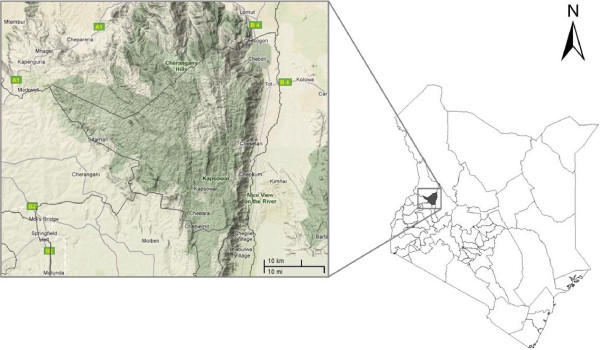
**Terrain map of Marakwet district on the left**, **and the Map of Kenya showing the position of Marakwet district in the right.** © 2010 Google - Map Data ©2010 Google, Tracks4Africa. Adapted from [23].

The study was conducted within Marakwet district, which is located in the North rift Valley region of Kenya (Figures 1, 2). The district, together with Keiyo, form Elgeyo Marakwet County [22]. The district is situated between 0° 51′ to 1° 19′ N and 35° 29′ to 35° 43′ E, and covers a total area of 1588 Km^2^ (Statoids). It has two regions; the highland also referred to by the residents as “*Mosop*”; and lowland located on the floor of Kerio Valley also known as “*Kew*” or “*Endo*”. In between the two regions is the escarpment, a long line of steep slopes consisting of cliffs separating the lowland and highland areas; with distinct vegetation type (Figure 2).

The highland region rises up to 3300 m above sea level at the highest point (Cheran’gany hills), and is mainly forested with low temperatures of about 13°C. The district is one of the most forested in the country, with natural forests covering 43% of the land, a total of 65, 000 hectares [24]. In addition, unlike other regions of Kenya such as the Mau forest, it has relatively not suffered heavy deforestation, and is a source of many streams which feed Lake Victoria in the Western part, and Lake Turkana in the East [23-25]. Cherangani Hills is part of the five main closed forests areas in Kenya that are protected by the government. The others are the Aberdares, Mount Kenya, Mount Elgon and South West Mau [26,27]. Although still heavily forested, Cherangani now faces risk of serious degradation due to illegal forest clearing [23,24,28]. The escarpment region is about 1000 m, and is part of the Great Rift Valley. It is mainly semi arid with temperatures rising up to 35°C.

The geographical landscape provides a uniquely wide topography, with a rich biodiversity comprising of different plant species, and in some instances largely undisturbed indigenous forests. The plant biodiversity in the Valley is particularly luxuriant and varied especially on the escarpment. Stunted trees, shrubs, succulents (*Sanseviera spp*.), and myriad of plants grow here in profusion. The herbalists use the numerous plants found throughout the diverse ecosystems from the Kerio Valley to the highlands [23,24]. Information on the most prevalent diseases within the district was obtained from the Medical officer of Health office. The population per doctor ratio in Elgeyo Marakwet County is 62,000: 1 [22,29].

### Data collection

The research team comprised of a group of professionals from both the medical field and plant specialists led by four scientists; a plant specialist (Kipkore), taxonomist (Wanjohi), a pharmacologist (Kigen) and an ophthalmologist (Rono). Apart from their respective professions, Kipkore and Kigen are residents of Elgeyo Marakwet County, and could therefore easily communicate in the local dialect. Rono has been involved in Trachoma control programs within the county, and is well known in the District. Ethnobotanical survey was conducted in different parts of Marakwet district over a 12-month period between February 2011 and January 2012.

Reconnaissance visits were initially conducted in order to identify the known/authentic herbalists in the district. The initial selection was based on the willingness of herbalists to voluntary give information and interaction with the researchers during consultative meetings. These meetings were participatory in nature, with acquaintances from the local communities as facilitators. Local opinion leaders including Church and village elders, Chiefs and Councilors were enlisted to assist in the identification, and to convince the herbalists to volunteer the information. Formal interviews were avoided. This was crucial as the herbalists had to clearly understand our motives before providing information, since most of the practicing herbalists were old and uneducated.

Ethnobotanical data was collected from the herbalists, both men and women practicing across the district. Their age ranged from 35 to 76 years, with the number of female herbalists being 74(66%) and their male counterparts 40(38%). The herbalists were interviewed within their practice, which in most cases was their residence, or Market centres. After that, the interviewees were requested to accompany the authors to the field to identify the plants. The plants were then photographed and collected. Where possible, the patients were also interviewed in order to corroborate the evidence of their treatment with the claims by the herbalists. The medicinal plants used in healthcare, herbal drugs preparations, local/botanical names; and the diseases treated were recorded. A total of 112 herbalists, all being Marakwets were identified, and all were included the study. The collected plants were identified by Kipkore and Wanjohi and all voucher specimens were deposited at the University of Eldoret Botanical Herbarium, for future reference. Kigen and Rono interviewed both the herbalists and patients, in order to identify the illnesses treated. Nomenclature of trees, shrubs and lianas were as per “Kenya Trees Shrubs and Lianas” [30], while the herbs were named according to “Kenya Upland Wild Flowers” [31]. The data was then compared to that from the previous studies that have been carried out in the region.

## Results and discussion

A total of 111 medicinal plants species were collected, out of which 3 could not be identified. The list of the plants and their respective uses are as outlined in Table 1. The 108 identified plants belonged to of 53 families. This compares well to previous studies that were done in Mt Elgon, where the other Kalenjin, the Sabaot reside [21], but significantly higher than that of their Nandi cousins [20]. This could be attributed to the diverse ecosystem in both Marakwet and Mt Elgon regions, and the fact that herbal practice is still widespread in the two districts.

**Table 1:** List of the various Herbal plants used by herbalistsand corresponding uses

***Botanical name***	***Voucher no.***	***Local name***	***Common name***	***Family***	***Habit***	***Habitat***	***Parts used***	***Method***	***General plant uses***
*Acacia brevispica* Harms	WBHG/13/081	*Korniswo*	*Prickly Thorn Bonsai*	*Mimosaceae*	*Shrub*	*Lowland*/*escarpment*	*Leaves*	*Crushed and rubbed on skin*	*Itchy skin rashes*
*Acacia brevispica* Harms	WBHG/13/081	*Korniswo*	*Prickly Thorn Bonsai*	*Mimosaceae*	*Shrub*	*Lowland*/*escarpment*	*Leaves*	*Crused and applied*	*Removal of ganglion*
*Acacia hockii* De. Willd.	WBHG/13/026	*Churur*	*Whistling thorn*/*shittim wood*	*Mimosaceae*	*Tree*	*Escarpment*	*Bark*	*Boiled and administered*	*Abdominal* (*colic*) *pains*
*Acacia hockii* De. Willd.	WBHG/13/026	*Churur*	*Whistling thorn*/*shittim wood*	*Mimosaceae*	*Tree*	*Escarpment*	*Bark*	*Boiled with water*	*Herbal* “*tea*”
*Acacia lahai* [Steud. & Hochst. ex] Benth	WBHG/13/038	*Seretetwo*	*Red thorn*	*Mimosaceae*	*Tree*	*Highland*	*Leaves*	*Pounded and mixed with other herbs*	*Synergistic herb*
*Acacia mellifera* (Vahl) Benth	WBHG/13/043	*Ngowo*	*Black thorn*/*wait*-*a*-*bit thorn*	*Mimosaceae*	*Shrub*	*Lowland*	*Roots*	*Boiled and administered*	*Sexually transmitted diseases*
*Acacia mellifera* (Vahl) Benth	WBHG/13/043	*Ngowo*	*Black thorn*/*wait*-*a*-*bit thorn*	*Mimosaceae*	*Shrub*	*Lowland*	*Higher parasites*	*Burnt and ashes licked*	*Rheumatism*
*Acacia nilotica* (L.) Delile	WBHG/13/068	*Ngopkwo*	*Thorntree*/*whistling thorn*	*Mimosaceae*	*Tree*	*Lowland*/*escarpment*	*Bark*	*Bark chewed when raw or boiled*	*Abdominal* (*colic*) *pains*
*Acacia tortilis* Hayne	WBHG/13/039	*Sesia*	*Shittim wood*	*Mimosaceae*	*Tree*	*Lowland*/*escarpment*	*Fruit bodies*	*Burnt and ash licked*	*Cancer*
*Acalypha fruticosa* Forsk.	WBHG/13/101	*Sawiyon*	*Birch*-*leaved Acalypha*	*Euphorbiaceae*	*Herb*	*Lowland*/*escarpment*	*Leaves*	*Crushed and applied on the site*	*Scorpion*/*bee stings*
*Acokanthera schimperi* (A. DC.) Benth. & Hook.	WBHG/13/110	*Kelyo*	*Arrow*-*poison plant*	*Apocynaceae*	*Tree*	*Escarpment*	*Roots*	*Boiled and applied*	*Arrow poison*
*Albizia spp*.	WBHG/13/063	*Seet*	*Flea tree* /*frywood*	*Mimosaceae*	*Tree*	*Escarpment*	*Fruit bodies*	*Burnt and ash licked*	*Cancer*
*Albizia spp*.	WBHG/13/063	*Seet*	*Flea tree* /*frywood*	*Mimosaceae*	*Tree*	*Escarpment*	*Bark*	*Boiled and administered*	*Abortifacient*, *Contraceptive*
*Albuca bracteata* (*Thunb*.) J.C. Manning & Goldblatt	WBHG/13/049	*K*’*dow*	*Pregnant onion plant*	*Asparagaceae*	*Herb*	*Lowland*	*Bulbs*	*Boiled and administered*	*Cancer*
*Aloe spp*.	WBHG/13/095	*Chepkenderetwo*	*Burn plant*	*Aloaceae*	*Herb*	*Lowland*/*escarpment*	*Leaves*	*Crushed and sap applied*	*Wounds*
*Aloe spp*.	WBHG/13/095	*Chepkenderetwo*	*Burn Plant*	*Aloaceae*	*Herb*	*Lowland*/*escarpment*	*Leaves*	*Sap applied on the operated area*	*Craniotomy* (*surgery*)
*Balanites aegyptiaca* (L.) Del.	WBHG/13/011	*Tuyunwo*	*Desert date*	*Balanitaceae*	*Tree*	*Lowland*/*escarpment*	*Leaves*, *fruits*	*Cooked as vegetable*	*Healing vegetable*
*Balanites rotundifolia* (Tiegh.) Blatt	WBHG/13/118	*Lomion*	*Not found*	*Balanitaceae*	*Tree*	*Lowland*/*escarpment*	*Fruits*	*Crush the fruits*, *soak in water and apply*	*Pesticide*
*Berchemia discolor* (Klotsch) Hemsl	WBHG/13/076	*Muchukwo*	*Bird plum*	*Rhamnaceae*	*Tree*	*Lowland*/*escarpment*	*Roots*	*Boiled and administered*	*Erectile dysfunction*
*Boscia coriacea* Pax.	WBHG/13/062	*Sorukwo*	*Not found*	*Capparidaceae*	*Tree*	*Lowland*/*escarpment*	*Seeds*	*Boiled and administered*	*Obesity*
*Bryophyta* spp.	WBHG/13/096	*Chebumbu*	*Green Moss*	*Oxallidaceae*	*Herb*	*Highland*/*escarpment*	*Whole plant*	*Burnt and ash licked*	*Heartburn*/*Teething in children*
*Bryophyta* spp.	WBHG/13/096	*Chebumbu*	*Green Moss*	*Oxallidaceae*	*Herb*	*Highland*/*escarpment*	*Whole plant*	*Burnt and ashes rubbed on the gums*	*To relive pain associated with teething in children*
*Calotropis procera* (Aiton) W.T. Aiton	WBHG/13/079	*Kibou*	*Giant milkweed*/*calotropis*	*Asclepiadaceae*	*Shrub*	*Lowland*	*Leaves*	*Burnt and ash administered*	*Emetic*
*Capparis cartilaginea* Decne.	WBHG/13/091	*Chepteretwo*	*Caper shrub*	*Capparidaceae*	*Shrub*	*Lowland*	*Leaves*	*Chewed*/ *burnt and ash licked*	*Heartburn*/*peptic ulcers*
*Capparis cartilaginea* Decne.	WBHG/13/091	*Chepteretwo*	*Caper shrub*	*Capparidaceae*	*Shrub*	*Lowland*	*Flowers*	*Dried*, *crushed and added to meat*	*Food flavour*
*Caralluma acutangula*	WBHG/13/031	*Mochontopokot*	*Caralluma*	*Asclepiadaceae*	*Herb*	*Lowland*	*Whole plant*	*Whole plant chewed*	*Chest congestion* (*wheezing*)
*Carissa edulis* (Forssk.) Vahl	WBHG/13/091	*Legetetwet*	*Egyptian carissa*	*Apocynaceae*	*Shrub*	*Highland*/*escarpment*/*lowland*	*Roots*	*Boiled and added to other herbs or used alone*	*General malaise*
*Carissa edulis *(Forssk.) Vahl	WBHG/13/091	*Legetetwet*	*Egyptian carissa*	*Apocynaceae*	*Shrub*	*Highland*/*escarpment*/*lowland*	*Fruits*	*Consumed in whole*	*Appetizer*
*Cassia didymobotrya* Fres.	WBHG/13/094	*Senetwo*	*Peanut*- *butter cassia*	*Caesalpiniaceae*	*Shrub*	*Highland*/*escarpment*	*Dried bark*	*Pound and applied on bleeding area*	*Antihaemorrhagic* (*stops bleeding*)
*Cassia didymobotrya* Fres.	WBHG/13/094	*Senetwo*	*Peanut*- *butter cassia*	*Caesalpiniaceae*	*Shrub*	*Highland*/*escarpment*	*Leaves*/*bark*	*Boiled and administered*	*Hypertension*
*Cassia obtusifolia* Linn *or Cassia occidentalis*L.	WBHG/13/070or 070a	*Kipkurkuris*	*Senna*	*Caesalpiniaceae*	*Herb*	*Lowland*/*escarpment*	*Leaves and seeds*	*Boiled and administered*	*Abortifacient*
*Cirsium vulgare* (Savi.) Ten	WBHG/13/013	*Tokoukowo*	*Spear thistle*	*Asteraceae*	*Herb*	*Highland*/*escarpment*	*Leaves*/*twigs*	*Burnt and ash licked*	*Heartburn*
*Cirsium vulgare* (Savi.) Ten	WBHG/13/013	*Tokoukowo*	*Spear Thistle*	*Asteraceae*	*Herb*	*Highland*/*escarpment*	*Leaves*/*Twigs*	*Burnt ash applied on incisions made on the skin*	*Talisman*
*Clerodendrum myricoides* (Hochst.) Vatke	WBHG/13/005	*Chebobet*	*Blue glory bower*	*Lamiaceae*	*Tree*	*Escarpment*	*Leaves*	*Burnt and ash licked*	“*Kipei*” *condition*
*Clutia abyssinica* Jaub & Spach	WBHG/13/089	*Kapkurelwo*	*Smooth*-*fruited lightning*-*bush*	*Euphorbiaceae*	*Shrub*	*Lowland*/*riverine*	*Roots*	*Boiled and mixed with other herbs*	*Synergistic herb*
*Clutia abyssinica* Jaub & Spach	WBHG/13/089	*Kapkurelwo*	*Smooth*-*fruited lightning*-*bush*	*Euphorbiaceae*	*Shrub*	*Lowland*/*riverine*	*Roots*	*Boiled and administered*	*Erectile dysfunction*
*Coccinia grandis* (L.) Voigt	WBHG/13/019	*Minjilwo*	*Ivy ground*	*Cucurbitaceae*	*Herb*	*Lowland*	*Leaves*	*Boiled and used as vegetable*	*Treatment of heavy snoring*
*Combretum apiculatum* Sond.	WBHG/13/111	*Leleiya*	*Red bushwillow*	*Combretaceae*	*Tree*	*Escarpment*	*Fruit bodies*	*Burnt and ash licked*	*Cancer*
*Commicarpus africanus* (*Lour*.) *Dandy*	WBHG/13/065	*Tanagit*	*Comicarp* (*Catalan*)	*Nyctaginaceae*	*Herb*	*Escarpment*	*Leaves*	*Crushed leaves rubbed on the ganglion*	*Removal of ganglion*
*Cordia sinensis* Lam.	WBHG/13/007	*Adomeyon*	*Grey*-*leaved saucer berry*	*Boraginaceae*	*Tree*	*Lowland*	*Twigs*	*Break tender twigs for use*	*Toothbrush*
*Crassocephalum mannii*	WBHG/13/042	*Terkekwo*	*Thickhead*	*Asteraceae*	*Shrub*	*Highland*/*escarpment*	*Roots*	*Boiled and administered*	*Arthritis*
*Crotalaria incana* L.	WBHG/13/060	*Kimira*	*Woolly Rattlepod*	*Fabaceae*	*Herb*	*Lowland*/*escarpment*	*Leaves*	*Boiled and used as vegetable*	*Anaemia*
*Crotalaria incana* L.	WBHG/13/060	*Kimira*	*Woolly Rattlepod*	*Fabaceae*	*Herb*	*Lowland*/*escarpment*	*Leaves*	*Cooked as vegetable*	*Healing vegetable*
*Croton ciliatoglandulifer* Ortega	WBHG/13/077	*Kibichan*	*Mexican croton*	*Euphorbiaceae*	*Liana*/*climber*	*Lowland*/*escarpment*	*Roots*	*Boiled and mixed with other herbs*	*Synergistic herb*
*Croton ciliatoglandulifer* Ortega	WBHG/13/077	*Kibichan*	*Mexican croton*	*Euphorbiaceae*	*Liana*/*climber*	*Lowland*/*escarpment*	*Roots*	*Chewed raw*/ *boiled with other herbs*	*Heartburn*
*Croton dichogamus* Pax	WBHG/13/064	*Kerelwo*	*Orange leavedcroton*	*Euphorbiaceae*	*Shrub*	*Escarpment*	*Higher parasites on this plant*	*Burnt and ash licked*	“*Kipei*” *condition* (*abdominal pain and oral thrush*)
*Croton dichogamus* Pax	WBHG/13/064	*Kerelwo*	*Orange leavedcroton*	*Euphorbiaceae*	*Shrub*	*Escarpment*	*Bark*, *roots and flowers*	*Boiled and administered*	*Chest congestion* (*wheezing*)
*Croton megalocarpus* Hutch.	WBHG/13/045	*Otonwet*	*Broadleaved croton*	*Euphorbiaceae*	*Tree*	*Escarpment*	*Roots*	*Boiled and administered*	*Purgative*
*Cucumisdipsaceu s* C. G. Ehrenb. ex Spac	*WBHG*/*13*/*055*	*Kisangwa*	*Bitter gourd*	*Cucurbitaceae*	*Herb*	*Lowland*/*escarpment*	*Fruits*	*Crushed and mixed with little water and administered to patient*	*Emetic*
*Cyperus esculentus* L.	WBHG/13/103	*Morgut*	*Yellow nutsedge*	*Cyperaceae*	*Herb*	*Lowland*/*swampy areas*	*Tubers*	*Chewed*	*Stomachache*
*Cyperus esculentus* L.	WBHG/13/103	*Morgut*	*Yellow nutsedge*	*Cyperaceae*	*Herb*	*Lowland*/*swampy areas*	*Tubers*	*Chewed*	*Abdominal* (*colic*) *pains*/*Talisman*
*Cyperus esculentus* L.	WBHG/13/103	*Morgut*	*Yellow nutsedge*	*Cyperaceae*	*Herb*	*Lowland*/*swampy areas*	*Tubers*	*Chewed or boiled*	*Colic pain in children*
*Cyperus esculentus* L.	WBHG/13/103	*Morgut*	*Yellow nutsedge*	*Cyperaceae*	*Herb*	*Lowland*/*swampy areas*	*Tubers*	*Chewed and applied*	*Talisman*
*Cyphostemma serpens* (Hochst. ex*A.Rich*.) Desc	WBHG/13/021	*Kirorot*	*Not found*	*Vitaceae*	*Herb*	*Lowland*/*escarpment*	*Dried stem*	*Boiled and administered*	*Abortifacient*
*Cyphostemma cyphopetalum* (Fresen.) Descoings	WBHG/13/075	*Kibungwach*	*Not found*	*Vitaceae*	*Herb*	*Escarpment*	*Tubers*	*Crush and apply on the pests*	*Pesticide*
*Dactylocteniuma egyptium* L.	WBHG/13/073	*Anyinya*	*Egyptian crowfoot grass*	*Poaceae*	*Herb*	*Lowland*/*escarpment*	*Leaves*	*Crushed and chewed raw*	*Heartburn*
*Dactylocteniuma egyptium* L.	WBHG/23/073	*Anyinya*	*Egyptian crowfoot grass*	*Poaceae*	*Herb*	*Lowland*/*escarpment*	*Whole plant*	*Cooked as vegetable*	*Healing vegetable*
*Digeramuricata* L.	*WBHG*/*13*/*099*	*Chesugut*	*False Amaranth*	*Amaranthaceae*	*Herb*	*Lowland*	*Leaves*	*Cooked as vegetable*	*Healing vegetable*
*Diospyros scabra* (Chiov.) Cufod	WBHG/13/016	*Turetwo*	*Ebony tree*	*Ebenaceae*	*Tree*	*Escarpment*	*Bark*	*Boil and patient bathed in concoction and covered*	*Fever in children*
*Diospyros scabra* (Chiov.) Cufod	WBHG/13/016	*Turetwo*	*Ebony tree*	*Ebenaceae*	*Tree*	*Escarpment*	*Twigs*	*Break tender twigs for use*	*Toothbrush*
*Dodonaea viscose* (L.) Jacq.	WBHG/13/098	*Tebelekwo*	*Hopbush*	*Sapindaceae*	*Shrub*	*Escarpment*	*Twigs*	*Break tender twigs and use*	*Toothbrush*
*Dolichos spp*.	WBHG/13/104	*Kipchoror*	*Hyacinth bean*	*Fabaceae*	*Herb*	*Lowland*	*Dry leaves*	*Pound*, *mixed with water and administered*	*Enhance lactation in breastfeeding mothers*
*Drimia indica* (Roxb.) Jessop	WBHG/13/046	*Barangoya*	*Indian*-*squill*	*Asparagaceae*	*Herb*	*Lowland*/*escarpment*	*Bulbs*	*Bulb applied on the ulcers*	*Cancer*
*Ehretia cymose var. silvatica* Guerke	WBHG/13/066	*Kabonbonet or Mororion*	*Stamperwood*	*Boraginaceae*	*Shrub*	*Highland*	*Bark*	*Boiled and administered*	*Stomachache*
*Ekebergia rueppelliana* Fres	WBHG/13/045	*Kerbu*	*Ekebergia*	*Meliaceae*	*Tree*	*Lowland*	*Bark*	*Boiled and administered*	*Purgative*
*Ensete ventricosum* (Welw.) E.E. Cheesman	WBHG/13/002	*Sosurwo*	*False banana*	*Musaceae*	*Shrub*	*Highland*/*escarpment*	*Tip end* (*flower*)	*Crushed*, *dried and burnt. Ash licked*	*Heartburn*
*Erythrina abyssinica* DC.	WBHG/13/029	*Korkorwo*	*Corpse flower*	*Fabaceae*	*Tree*	*Highland*	*Bark*	*Boiled and administered*	*Mumps*
*Euclea divinorum* Hiern.	WBHG/13/052	*Uswo*	*Magic Guarri*	*Ebenaceae*	*Shrub*	*Escarpment*	*Twigs*	*Break tender twigs for use*	*Toothbrush*
*Eucleadivinorum*Hiern.	WBHG/13/052	*Uswo*	*Magic Guarri*	*Ebenaceae*	*Shrub*	*Escarpment*	*Bark*	*Pound and applied on the incision made on the bitten site*	*Antivenom*
*Faidherbia albida* (Delile) A.Chev.	WBHG/13/027	*Kokocha*	*Apple ring acacia*	*Mimosaceae*	*Tree*	*Lowland*/*riverine*/*swampy areas*	*Fruit bodies*	*Burnt and ash licked*	*Cancer*
*Faurea saligna* Harvey	WBHG/13/018	*Sirirte*	*Beechwood*	*Proteaceae*	*Tree*	*Highland*	*Bark*	*Boiled and administered*	*Period pains*
*Flacourtia indica* (Burm.f.) Merr.	WBHG/13/072	*Tingoswo*	*Governor*’*s plum*	*Flacourtiaceae*	*Tree*	*Lowland*/*escarpment*	*Fruits*	*Dry the fruit and use*	*Fermentation*
*Fuerstiaafricana* T.C.E. Fries	WBHG/13/004	*Kipirirwo*	*Not found*	*Lamiaceae*	*Herb*	*Escarpment*	*Leaves*	*Crushed and sap instilled*	*Eye ailments*
*Fuerstia africana* T.C.E. Fries	WBHG/13/004	*Kipirirwo*	*Not found*	*Lamiaceae*	*Herb*	*Escarpment*	*Leaves*	*Crushed and sap applied*	*Toothache*
*Gardenia sp*	WBHG/13/044	*Mogilion*/*Kobilwo*	*Forest gardenia*	*Rubiaceae*	*Tree*	*Lowland*	*Fruits*	*Crushed and administered to patient*	*Emetic*/*purgative*
*Grewia villosa* Willd	WBHG/13/020	*Mokurwo*	*Mallow raisin*	*Tiliaceae*	*Shrub*	*Lowland*	*Leaves*	*Cover milk gourd with it*	*Fermentation*
*Gynandropsis gynandra* (L.) Briq.	WBHG/13/025	*Sachan*	*African spider flower*	*Capparidaceae*	*Herb*	*Lowland*/*escarpment*	*Leaves*	*Cooked and used as vegetable*	*Healing vegetable*
*Heeria reticulata* (Baker f.) Engl	WBHG/13/008	*Mutung*’*wo*	*Raisin bush*	*Anacardiaceae*	*Tree*	*Escarpment*	*Bark*	*Burnt and ash licked*	*Removal of retained placenta in both human and animals*
*Heeria reticulata* (Baker f.) Engl	WBHG/13/008	*Mutung*’*wo*	*Raisin bush*	*Anacardiaceae*	*Tree*	*Escarpment*	*Leaves*	*Crush*, *soak in water and apply*	*Constipation in animals*
*Hoslundia opposite* Valh.	WBHG/13/083	*Sumeyon*	*Orange bird berry*	*Lamiaceae*	*Shrub*	*Lowland*/*escarpment*	*Leaves*, *roots*	*Boiled and administered*	*Colic pain in children*
*Hoslundia opposite* Valh.	WBHG/13/083	*Sumeyon*	*Orange bird berry*	*Lamiaceae*	*Shrub*	*Lowland*/*escarpment*	*Roots*, *leaves*	*Boiled and administered*	*Amoebiasis*
*Hoslundia opposite* Valh.	WBHG/13/083	*Sumeyon*	*Orange bird berry*	*Lamiaceae*	*Shrub*	*Lowland*/*escarpment*	*Leaves*	*Crushed*, *soaked in water and gargled*	*Oral thrush*
*Hypoestes forskaolii* (Vahl) Roem. & Schult.	WBHG/13/059	*Sirkonwo*/*Kaberkewo*	*White ribbon bush*	*Acanthaceae*	*Shrub*	*Lowland*/*escarpment*	*Leaves* , *twigs*	*Crush*, *soak in water and apply*	*Pesticide*
*Iboza spp*.	WBHG/13/012	*Lonwo*	*Nutmeg bush*	*Lamiaceae*	*Shrub*	*Escarpment*	*leaves*	*Crushed and pound*	*Perfume*
*Indigofera arrecta* L.	WBHG/13/034	*Sargellat*	*African indigo*	*Fabaceae*	*Herb*	*Lowland*/*escarpment*	*Roots*	*Boiled and mixed with other herbs*	*Synergistic herb*
*Indigofera arrecta* L	WBHG/13/034	*Sargellat*	*African indigo*	*Fabaceae*	*Herb*	*Lowland*/*escarpment*	*Roots*	*Boiled and administered*	*Stomachache*
*Indigofera arrecta* L	WBHG/13/034	*Sargellat*	*African indigo*	*Fabaceae*	*Herb*	*Lowland*/*escarpment*	*Roots*	*Boiled & mixed with other herbs*	*Cancer*
*Indigofera arrecta* L	WBHG/13/034	*Sargellat*	*African indigo*	*Fabaceae*	*Herb*	*Lowland*/*escarpment*	*Roots*	*Chewed*	*Toothache*
*Ipomoea lapidosa*Vatke	WBHG/13/078	*Kimugugu*	*Common morning glory*	*Convolvulaceae*	*Liana*/*climber*	*Lowland*/*escarpment*	*Stem*	*Boiled and administered*	*Sexually transmitted diseases*
*Justica spp*.	WBHG/13/024	*Kepkalomion*	*Jacobinia*	*Acanthaceae*	*Herb*	*Escarpment*	*Leaves*	*Boiled and administered*	*Abdominal* (*colic*) *pains*
*Kalanchoe germanae* Raym.-*Hame*t ex Raadts	WBHG/13/030	*Kibarbany*	*Air plant*	*Crassulaceae*	*Herb*	*Lowland*/*escarpment*	*Leaves*	*Pound and rubbed on ganglion area*	*Removal of ganglion*
*Kalanchoe germanae* Raym.-*Hame*t ex Raadts	WBHG/13/030	*Kibarbany*	*Air plant*	*Crassulaceae*	*Herb*	*Lowland*/*escarpment*	*Leaves*	*Pound and rubbed on painful area*	*Poultice*
*Kigelia Africana* (Lam.) Beneth	WBHG/13/048	*Rotion*	*African sausage*	*Bignoniaceae*	*Tree*	*Lowland*/*escarpment*	*Fruits*	*Split and dry*	*For fermentation during the brewing of traditional beer*
*Lannea fulva* (Engl.) Engl.	WBHG/13/056	*Lolotwo*	*Not found*	*Anacardiaceae*	*Tree*	*Lowland*/*escarpment*	*Bark*	*Pound and mixed with other herbs*	*Antivenom*
*Lantana trifolia* L.	WBHG/13/046	*Bekaptarit*	*Three*-*leafshrubverbena*	*Verbenaceae*	*Shrub*	*Lowland*/*escarpment*	*Leaves*/*twigs*	*Boiled and administered*	*Abdominal* (*colic*) *pains*
*Lantana trifolia* L.	WBHG/13/046	*Bekaptarit*	*Three*-*leafshrubverbena*	*Verbenaceae*	*Shrub*	*Lowland*/*escarpment*	*Leaves*, *twigs & fruits*	*Boiled and administered*	*Enhance lactation in breastfeeding mothers*
*Leptadenia hastate* (Pers.) Decne.	WBHG/13/058	*Kipchegin*	*Not found*	*Apocynaceae*	*Liana*/*climber*	*Lowland*/*escarpment*	*Tender leaves*	*Cooked as vegetable*	*Healing vegetable*
*Lippia javanica* (Burm. f.) Spreng	WBHG/13/053	*Chebokobil*	*Zinziba plant*	*Verbenaceae*	*Shrub*	*Highland*	*Leaves and twigs*	*Boiled and administered*	*Amoebiasis*
*Lippia javanica* (Burm. f.) Spreng	WBHG/13/053	*Chebokobil*	*Zinziba plant*	*Verbenaceae*	*Shrub*	*Highland*	*Leaves and twigs*	*Boiled together with maize*, *cassava*, *groundnuts and even tea*	*Food flavour*
*Maesa lanceolata* Forsk.	WBHG/13/087	*Mborion*	*False assegai*	*Myrsinaceae*	*Liana*/*climber*	*Lowland*	*Whole plant*	*Crush* , *soak in water and apply*	*Veterinary use as antipoison*
*Nuxia congesta* Fres	WBHG/13/082	*Chorwa*	*Brittle wood*	*Loganiaceae*	*Tree*	*Highland*	*Roots*	*Boiled and administered*	*Abdominal* (*colic*) *pains* /*Flu*
*Nuxia congesta* Fres	WBHG/13/082	*Chorwa*	*Brittle wood*	*Loganiaceae*	*Tree*	*Highland*	*Roots*	*Boiled and administered*	*Flu*
*Ocimum basilicum* L.	WBHG/13/028	*Klachir*	*Basil*	*Lamiaceae*	*Herb*	*Lowland*	*Leaves*	*Crushed and sap instilled on the affected eye*	*Eye ailments*
*Olea Africana* Mill.	WBHG/13/032	*Remit*	*Wild olive*	*Oleaceae*	*Tree*	*Highland*/*escarpment*	*Dried bark*	*Pound and powder applied*	*Eye ailments*
*Olea Africana* Mill.	WBHG/13/032	*Remit*	*Wild olive*	*Oleaceae*	*Tree*	*Highland*/*escarpment*	*Bark*	*Boiled and administered*	*Itchy rashes*
*Ornithogalum tenuifolium* Delaroche	WBHG/13/050	*Katagwa*	*Sea*-*onion*	*Hyacinthaceae*	*Herb*	*Lowland*/*escarpment*	*Tubers*	*Crushed and applied on joints*	*Arthritis*
*Pappea capensis* Eckyl & Zeyh	WBHG/13/023	*Kibiryokwo*	*Jacket plum*	*Sapindaceae*	*Tree*	*Lowland*/*escarpment*	*Fruit bodies*	*Burnt and ash licked*	*Cancer*
*Pappea capensis* Eckyl & Zeyh	WBHG/13/023	*Kibiryokwo*	*Jacket plum*	*Sapindaceae*	*Tree*	*Lowland*/*escarpment*	*Higher parasites*	*Burnt and ashes licked*	*Rheumatism*
*Pavetta abyssinica* Fresen	WBHG/13/107	*Cheptabirbirwo*	*Not found*	*Rubiaceae*	*Shrub*	*Lowland*/*escarpment*	*Bark*	*Crushed and administered*	*Purgative*
*Pentas longiflora* W.R.B. Oliv.	WBHG/13/093	*Chebirbirgorok*	*Not found*	*Rubiaceae*	*Shrub*	*Escarpment*	*Fruits*/*bark*	*Boiled and administered*	*Malaria like symptoms*
*Periploca linearifolia* Dill. & Rich.	WBHG/13/073	*Sinende*	*Not found*	*Apocynaceae*	*Liana*/*climber*	*Highland*	*Fruits*/*Leaves*		*Ceremonial plant*
*Periploca linearifolia* Dill. & Rich.	WBHG/13/073	*Sinende*	*Not found*	*Apocynaceae*	*Liana*/*climber*	*Highland*	*Fruits*/*Leaves*	*Crush the fruits*, *soak in water and apply*	*Pesticide*
*Podocarpus graciliar* Pilger	WBHG/13/067	*Bennet*	*African fern tree*/*bastard yellow wood*	*Podocarpaceae*	*Tree*	*Highland*	*Bark*	*Boiled and administered*	*Hypertension*
*Portulaca quadrifida* L.	*WBHG*/*13*/*037*	*Kitumerio*	*Small*-*leaved purslane*	*Portulacaceae*	*Herb*	*Lowland*	*Whole plant*	*Cooked as vegetable*	*Healing vegetable*
*Portulaca oleracea* L.	WBHG/13/092	*Chemorin*	*Asthma weed*	*Portulacaceae*	*Herb*	*Lowland*	*Whole crushed plant*	*Boiled with other herbs*	*Cancer*
*Prunus africana* (Hook f.) Kalkm.	WBHG/13/014	*Tendwo*	*African plum tree*	*Rosaceae*	*Tree*	*Highland*	*Bark*	*Boiled and administered*	*Hypertension*
*Prunus Africana* (Hook f.) Kalkm.	WBHG/13/014	*Tendwo*	*African plum tree*	*Rosaceae*	*Tree*	*Highland*	*Bark*/*roots*	*Boiled and administered*	*Enlarged prostate*
*Psiadia arabica* Jaub.et Spach	WBHG/13/047	*Konocho*	*Not found*	*Asteraceae*	*Shrub*	*Escarpment*	*Bark*	*Boiled with water*	*Herbal* “*tea*”
*Ricinus communis* L.	WBHG/13/017	*Menwa*	*Castor bean*	*Euphorbiaceae*	*Shrub*	*Lowland*/*escarpment*	*Seeds*	*Crushed and oil applied*	*Treatment of hides and skins*
*Rumex acetosella L*.	WBHG/13/080	*Kibongbong*	*Sheep sorrel*	*Polygonaceae*	*Shrub*	*Highland*/*escarpment*	*Tubers*	*Chewed or boiled and administered*	*Hypertension*
*Rumex acetosella L*	WBHG/13/080	*Kibongbong*	*Sheep sorrel*	*Polygonaceae*	*Shrub*	*Highland*/*escarpment*	*Tubers*	*Chewed or boiled and administered*	*Diabetes*
*Saba comorensis* (B*ojer e*x A.DC.) Pichon	WBHG/13/112	*Ochon*	*Rubber vine*	*Apocynaceae*	*Liana*/*climber*	*Lowland*/*escarpment* (*riverine*)	*Fruits*	*Fruit consumed*	*Diagnosis of enlarged prostate*
*Salvadora persica* Wall.	WBHG/13/033	*Chekowo*	*Toothbrush tree*	*Salvadoraceae*	*Tree*	*Lowland*/*escarpment*	*Twigs*	*Break tender twigs and use*	*Toothbrush*
*Sansevieria intermedia*	WBHG/13/009	*Sorogat*	*Mother*-*in*-*law tongue*	*Agavaceae*	*Herb*	*Lowland*/*escarpment*	*Leaves*	*Sap used as bait*	*Used to kill snakes*
*Schefflera volkensii* (Engl.) Harms	WBHG/13/088	*Tingwon*	*Schefflera*	*Araliaceae*	*Tree*	*Highland*	*Dried resin*	*Resin sniffed*	*Inhaled to clear blocked nose*
*Schefflera volkensii* (Engl.) Harms	WBHG/13/088	*Tingwon*	*Schefflera*	*Araliaceae*	*Tree*	*Highland*	*Dried resin*	*Smear on body*	*Perfume*
*Sclerocarya birrea* (A. Rich.) Hochst	WBHG/13/106	*Orolwo*	*Amarula tree*	*Anacardiaceae*	*Tree*	*Lowland*/*escarpment*	*Bark*	*Chewed or boiled and administered*	*Diabetes*
*Solanum incanum* L.	WBHG/13/035	*Kalobotwo*	*Thorn Apple*	*Solanaceae*	*Shrub*	*Lowland*/*escarpment*	*Roots*	*Boiled or chewed raw*	*Abdominal* (*colic*) *pains*
*Solanum incanum* L.	WBHG/13/035	*Kalobotwo*	*Thorn Apple*	*Solanaceae*	*Shrub*	*Lowland*/*escarpment*	*Roots*	*Boiled or chewed*	*Colic pain in children*
*Sphaeranthus ukambensis Vatke & O.Hoffm*	WBHG/13/085	*Moyon*	*Not found*	*Asteraceae*	*Shrub*	*Lowland*	*Roots*	*Chewed or boiled and administered*	*Stomachache resulting food poisoning*
*Spilanthes mauritiana* (Pers.) D.C	WBHG/13/090	*Kibutkut*	*Not found*	*Asteraceae*	*Herb*	*Highland*/*escarpment*/*lowland*	*Whole plant*	*Crushed and applied on the affected tooth*	*Toothache*
*Spilanthes mauritiana* (Pers.) D.C	WBHG/13/090	*Kibutkut*	*Not found*	*Asteraceae*	*Herb*	*Highland*/*escarpment*/*lowland*	*Whole crushed plant*	*Crushed and used as mouthwash*	*Oral thrush*
*Spilanthes mauritiana* (Pers.) D.C	WBHG/13/090	*Kibutkut*	*Not found*	*Asteraceae*	*Herb*	*Highland*/*escarpment*/*lowland*	*Whole plant*	*Crushed and applied on the area to be operated before surgery*	*Craniotomy* (*surgery*)
*Sterculia Africana* (Lou.r) Fiori	WBHG/13/086	*Ililwo*	*African star chestnut tree*	*Sterculiaceae*	*Tree*	*Lowland*/*escarpment*	*Seeds*	*Chewed*	*Erectile dysfunction*
*Terminalia brownie* Fresen	WBHG/13/069	*Koloswo*	*Mbarao* (*in Swahili*)	*Combretaceae*	*Tree*	*Escarpment*	*Bark*	*Boiled or chewed raw*	*Abdominal* (*colic*) *pains*
*Terminalia brownie* Fresen	WBHG/13/069	*Koloswo*	*Mbarao* (*in Swahili*)	*Combretaceae*	*Tree*	*Escarpment*	*Bark*	*Chewed or boiled and administered*	*Jaundice*
*Terminalia spinosa* Engl.	WBHG/13/092	*Kitong*’*wo*	*Spiny cluster*-*leaf*	*Combretaceae*	*Tree*	*Lowland*/*escarpment*	*Bark*	*Boiled and administered*	*Malaria like symptoms*
*Tragiabrevipes* Pax.	WBHG/13/069	*Kimelei*	*Shortspikenoseburn*	*Euphorbiaceae*	*Liana*/*climber*	*Lowland*/*escarpment*	*Roots*	*Pound and mixed with other herbs*	*Antivenom*
*Tribulusterrestris* L.	WBHG/13/001	*Kilesan*	*Bullhead*	*Zygophylaceae*	*Herb*	*Lowland*	*Whole crushed plant*	*Chewed or boiled and administered*	*Erectile dysfunction*
*Tribulusterrestris* L.	WBHG/13/001	*Kilesan*	*Bullhead*	*Zygophylaceae*	*Herb*	*Lowland*	*Whole plant*	*Cooked as vegetable*	*Healing vegetable*
*Uvariasp var. scheffleri*	WBHG/13/010	*Murkuiyo*	*Not found*	*Annonaceae*	*Shrub*/*climber*	*Lowland*/*escarpment*	*Roots*	*Boiled and administered*	*Common colds*/*cough*
*Vangueria apiculata* K. Schum.	WBHG/13/003	*Tabirirwo* (*Komolwo ne mining*)	*Triangle*-*flowered Wild*-*medlar*	*Rubiaceae*	*Shrub*	*Highland*/*escarpment*	*Fruits*	*Cooked as porridge and consumed*	*Food supplement*
*Vangueria madagascariensis* Gmel.	WBHG/13/041	*Komolwo neo*	*Common wild medlar*	*Rubiaceae*	*Shrub*	*Highland*/*escarpment*/*lowland*	*Fruits*	*Cooked as porridge and consumed*	*Food supplement*
*Vernoniabrachycalyx *O. Hoffm.	WBHG/13/074	*Kimagoi or Chebongony*	*Ironweed*	*Asteraceae*	*Herb*	*Lowland*	*Roots*	*Boiled and decoction administered*	*Emetic*
*Warburgia ugandensis* Sprague	WBHG/13/109	*Sokwon*	*East African green wood*	*Canellaceae*	*Tree*	*Highland*/*escarpment*	*Leaves*	*Chewed or soaked in hot water and administered*	*Stomachache*
*Warburgia ugandensis* Sprague	WBHG/13/109	*Sokwon*	*East African green wood*	*Canellaceae*	*Tree*	*Highland*/*escarpment*	*Bark*	*Burnt and smoked sniffed*	*Headache*
*Warburgia ugandensis* Sprague	WBHG/13/109	*Sokwon*	*East African green wood*	*Canellaceae*	*Tree*	*Highland*/*escarpment*	*Leaves*	*Crush and applied*	*Toothache*
*Warburgia ugandensis* Sprague	WBHG/13/109	*Sokwon*	*East African green wood*	*Canellaceae*	*Tree*	*Highland*/*escarpment*	*Leaves*	*Chewed or soaked in water and gargled*	*Common colds*/*cough*/*sore throat*
*Warburgia ugandensis* Sprague	WBHG/13/109	*Sokwon*	*East African green wood*	*Canellaceae*	*Tree*	*Highland*/*escarpment*	*Leaves*	*Soaked in water and gargled*	*Oral thrush*
*Withaniasomnifera* (L.) Dunal	WBHG/13/054	*Tarkukai*	*Red Cherry*	*Solanaceae*	*Shrub*	*Lowland*/*escarpment*	*Roots*/*leaves*	*Boiled and administered*	*Amoebiasis*
*Withania somnifera* (L.) Dunal	WBHG/13/054	*Tarkukai*	*Red Cherry*	*Solanaceae*	*Shrub*	*Lowland*/*escarpment*	*Leaves*	*Crushed and applied*	*Chronic skin ulcers*
*Ximenia Americana* L.	WBHG/13/105	*Kunyotwo*	*Yellow plum*	*Olacaceae*	*Tree*	*Lowland*/*escarpment*	*Seeds*	*Crushed and oil applied*	*Wounds*
*Ximenia Americana* L.	WBHG/13/105	*Kunyotwo*	*Yellow Plum*	*Olacaceae*	*Tree*	*Lowland*/*escarpment*	*Seeds*	*Crushed and oil applied*	*Treatment of hides and skins*
*Zanthoxylum chalybeum* Engl	WBHG/13/061	*Kochon*	*Knob wood*	*Rutaceae*	*Tree*	*Lowland*/*escarpment*	*Bark*/*seeds*	*Pounded together and mixed with other herbs*	*Synergistic herb*
*Zanthoxylum chalybeum* Engl	WBHG/13/061	*Kochon*	*Knob wood*	*Rutaceae*	*Tree*	*Lowland*/*escarpment*	*Bark*/*seeds*	*Boiled or chewed*	*Amoebiasis*
*Zanthoxylun chalybeum* Engl	WBHG/13/061	*Kochon*	*Knob wood*	*Rutaceae*		*Lowland*/*escarpment*	*Bark*	*Boiled and administered*	*Malaria like symptoms*
*Zanthoxylun chalybeum* Engl	WBHG/13/061	*Kochon*	*Knob wood*	*Rutaceae*	*Tree*	*Lowland*/*escarpment*	*Bark*	*Boiled and administered*	*Administered to recuperating patients* (*after surgery*)
*Zanthoxylun chalybeum* Engl	WBHG/13/061	*Kochon*	*Knob wood*	*Rutaceae*	*Tree*	*Lowland*/*escarpment*	*Higher parasites*	*Burnt and ashes licked*	*Rheumatism*
*Zehneria scabra* (Linn f.) Sond	WBHG/13/097	*Cheserya*	*Not found*	*Cucurbitaceae*	*Shrub*/*climber*	*Highland*	*Leaves*	*Crushed and administered*	*Common colds*/*cough*
*Zehneria scabra* (Linn f.) Sond	WBHG/13/097	*Cheserya*	*Not found*	*Cucurbitaceae*	*Shrub*/*climber*	*Highland*	*Whole plant*	*Boiled with other herbs*	*Cancer*
*Zehneria scabra* (Linn f.) Sond	WBHG/13/097	*Cheserya*	*Not found*	*Cucurbitaceae*	*Shrub*/*climber*	*Highland*	*Whole plant*	*Boiled and administered*	*Administered to recuperating patients*
*Ziziphus mauritania var. spinachristi* (L.) Wild.	WBHG/13/100	*Tilomwo*	*Christ thorn*/*jujube*	*Rhamnaceae*	*Tree*	*Lowland*	*Bark*	*Boiled with water*	*Herbal* “*tea*”
*Ziziphus mauritiana var. spina Christi* (L.) Willd	WBHG/13/100	*Tilomwo*	*Christ thorn*/*jujube*	*Rhamnaceae*	*Tree*	*Lowland*/*escarpment*	*Bark*	*Chewed raw*	*Abdominal* (*colic*) *pains*
*Unidentified*	WBHG/13/015	*Mindililwo ne mining*			*Herb*	*Escarpment*	*Leaves*	*Smoked*	*Drug of abuse*
*Unidentified*	WBHG/13/048	*Seremwo*			*Tree*	*Escarpment*	*Bark*	*Boiled and administered*	*Appetizer*
*Unidentified*	WBHG/13/057	*Turesio*			*Tree*	*Highland*	*Bark*/*roots*	*Burnt and with other herbs*	*Cancer*
*Unidentified*	WBHG/13/057	*Turesio*			*Tree*	*Highland*	*Leaves*	*Burnt and ashes licked*	“*Kipei*” *condition*

The family with the highest number of reported medicinal plant species was Mimosaceae 8(7.4%), followed by Euphorbiaceae 7(6.5%) and Asteraceae, 6(5.6%) (Table 2). Mt Elgon study had Fabaceae, Euphorbiaceae and Asteraceae while Nandi had Acanthaceae, Asteraceae and Amaranthaceae in the same order [20,21]. Of the three unidentified plants, we were informed that “*Turesio*” which is a large tree, has been overharvested for making cooking sticks and building poles. We also got to learn that “*Seremwo*”, also a tree grows in areas of the escarpment that are difficult to access, while “*Mindililwo ne mining*” is an ephemeral that only grows for a short period during the rainy season. Most of the medicinal plants used either grew on the lowlands or escarpment region. Table 3 shows the distribution of the plants as per region. Trees were most widely used plant parts 41(37%) followed by herbs 32(28%) and shrubs 29(26%). Only nine species (8%) were climbers (both liana and shrubs) (Table 4). All the plants were reported by their local names.

**Table 2:** Diversity of medicinal plant use

	**Family**	**No. of medicinal plant species**	**%**
1.	Mimosaceae	8	7.4
2.	Euphorbiaceae	7	6.5
3.	Asteraceae	6	5.6
4.	Lamiaceae	5	4.6
5.	Apocynaceae	5	4.6
6.	Rubiaceae	5	4.6
7.	Fabaceae	4	3.7
8.	Cucurbitaceae	3	2.8
9.	Capparidaceae	3	2.8
10.	Combretaceae	3	2.8
11.	Anacardiaceae	3	2.8
12.	Ebenaceae	2	1.9
13.	Olacaceae	2	1.9
14.	Solanaceae	2	1.9
15.	Verbenaceae	2	1.9
16.	Caesalpiniaceae	2	1.9
17.	Rhamnaceae	2	1.9
18.	Sapindaceae	2	1.9
19.	Acanthaceae	2	1.9
20.	Asclepiadaceae	2	1.9
21.	Asparagaceae	2	1.9
22.	Balanitaceae	2	1.9
23.	Boraginaceae	2	1.9
24.	Vitaceae	2	1.9
25.	Portulacaceae	2	1.9
26.	Canellaceae	1	0.9
27.	Rutaceae	1	0.9
28.	Cyperaceae	1	0.9
29.	Aloaceae	1	0.9
30.	Araliaceae	1	0.9
31.	Crassulaceae	1	0.9
32.	Loganiaceae	1	0.9
33.	Oxallidaceae	1	0.9
34.	Polygonaceae	1	0.9
35.	Rosaceae	1	0.9
36.	Zygophylaceae	1	0.9
37.	Agavaceae	1	0.9
38.	Annonaceae	1	0.9
39.	Bignoniaceae	1	0.9
40.	Convolvulaceae	1	0.9
41.	Flacourtiaceae	1	0.9
42.	Hyacinthaceae	1	0.9
43.	Meliaceae	1	0.9
44.	Musaceae	1	0.9
45.	Myrsinaceae	1	0.9
46.	Nyctaginaceae	1	0.9
47.	Podocarpaceae	1	0.9
48.	Proteaceae	1	0.9
49.	Salvadoraceae	1	0.9
50.	Amaranthaceae	1	0.9
51.	Poaceae	1	0.9
52.	Sterculiaceae	1	0.9
53.	Tiliaceae	1	0.9
		108	

**Table 3:** Plant habitat

	**No of species**	**%**
Lowland/escarpment	42	38
Escarpment	21	19
Lowland	20	18
Highland	12	11
Highland/escarpment	9	8
Highland/escarpment/lowland	3	3
Lowland/escarpment (riverine)	1	1
Lowland/riverine	1	1
Lowland/riverine/swampy areas	1	1
Lowland/swampy areas	1	1
	111	

**Table 4:** Plant habit

**Type**	**No of species**	**%**
Tree	41	37
Herb	32	29
Shrub	29	26
Liana/climber	7	6
Shrub/climber	2	2
	111	

Most treatment regimen included a combination of several herbal preparations from different plants. Emetics and purgatives were widely used as part of the treatment for several ailments. The leaves of *Acacia lahai*, barks and seeds of *Zanthoxylum chalybeum* and roots of *Indigofera arrecta*, *Croton ciliatoglandulifer*, *Clutia abyssinica* are combined with most of the preparations in order to improve the efficacy. The most commonly used medicinal plants were *Warburgia ugandensis* and *Zanthoxylum chalybeum* which were both used in the treatment of five different conditions. *Indigofera arrecta* was used in the treatment of four conditions. Among the species, Acacia was the most widely used with a total of six species employed in the treatment of various disorders. These include: *A. lahai*, *A. brevispica*, *A. hockii*, *A. mellifera* and *A. nilotica*. Among the diseases, cancer had the largest number of species used (10) followed by abdominal pains (9). Several plant species were also used as food supplements or to assist in recovery (Table 5).

**Table 5:** Medicinal plant uses

	**No of species used**	**Percentage**
Cancer	10	7.1
Abdominal (colic pains)	9	6.4
Food supplements	5	3.6
Heartburns	5	3.6
Herbs used by convalescing patients	5	3.6
Synergistic herbs	5	3.6
Amoebiasis	4	2.9
Emetics	4	2.9
Hypertension	4	2.9
Malaria like symptoms	4	2.9
Purgatives	4	2.9
Stomachache	4	2.9
Toothache	4	2.9
Abortifacients	3	2.1
Antivenom	3	2.1
Colic pain in children	3	2.1
Erectile dysfunction	3	2.1
Eye ailments	3	2.1
Food flavours	3	2.1
Herbal tea	3	2.1
Oral thrush	3	2.1
Removal of ganglions	3	2.1
“*Kipei*” condition	2	1.4
Administered after surgery	2	1.4
Arthritis	2	1.4
Chest congestion (wheezing)	2	1.4
Common colds/cough	2	1.4
Craniotomy (surgery)	2	1.4
Diabetes	2	1.4
Enhance lactation in breastfeeding mothers	2	1.4
Itchy skin rashes	2	1.4
Rheumatism	2	1.4
Sexually transmitted diseases	2	1.4
Wounds	2	1.4
Anaemia	1	0.7
Antihaemorrhagic (arrests bleeding)	1	0.7
Appetizers	1	0.7
Blocked nose	1	0.7
Chronic skin ulcers	1	0.7
Common colds/cough/sore throat	1	0.7
Diagnosis of an enlarged prostate	1	0.7
Enlarged prostate	1	0.7
Fever in children	1	0.7
Flu	1	0.7
General Malaise	1	0.7
Headache	1	0.7
Jaundice	1	0.7
Mumps	1	0.7
Obesity	1	0.7
Peptic ulcers	1	0.7
Period pains	1	0.7
Poultice	1	0.7
Removal of retained placenta	1	0.7
Stomachache resulting from food poisoning	1	0.7
Teething in children	1	0.7
Treatment of heavy snoring	1	0.7

Women herbalists mainly treated malaria, diarrhoeal diseases, children’s diseases and fertility, including erectile dysfunction and abortion. Men tended to specialize in hypertension, skin diseases, rheumatism/arthritis, and surgical procedures including craniotomy, removal of ganglions and setting fractures. The most prevalent infections on the highland areas were respiratory diseases mainly; common colds, flu, pneumonia, upper respiratory tract infections and allergies due to cold weather. Malaria and animal diseases are more prevalent in the lowlands areas. Other common diseases in the district include diarroheal diseases, skin diseases, eye diseases and urinary tract infections. The plants used by the herbalists in the two regions correlated to the prevalent diseases. For instance, common colds and flu were treated by highland plants such as *Schefflera volkensii*, *Zehneria scabra and Nuxia congesta*; whereas malaria was treated by either lowland or escarpment plants such as *Terminalia spinosa*, *Zanthoxylun chalybeum*, *Pentas longiflora* and *Diospyros scabra* (Table 1).

### Herbal preparations

The preparations consisted of roots, barks, leaves, twigs, sap and fruits and were prepared in different forms depending on the intended medicinal use. The proportions of the parts used are as illustrated in Table 6. Leaves were the most widely used (30%), followed by bark (21%) roots (16%), fruits (8%), whole plant (7%), seeds (5%), tubers (5%), fruit bodies (3%), higher parasites (2%) and flowers (2%).

**Table 6:** Proportions of plant parts used

**Part**	**%**
Leaves	30
Bark	21
Roots	16
Fruits	8
Whole plant	7
Seeds	5
Tubers	5
Fruit bodies	3
Higher parasites	2
Flowers	2

#### Decoctions

The plant parts are boiled or simply soaked in water and the decoction taken alone, or in some instances combined with honey, soup, or milk if the decoction is from a bitter plant. The soup is made from the head, intestines and hooves of an animal, preferably a goat or cow. A mixture of barks, leaves and fruits from several herbs may also be used depending on the condition being treated and the concoction administered to the patient.

#### Ashes

Leaves are dried and burnt to form powder ash locally referred to as “*Tusan*” in Marakwet. The ashes may then be licked, or in some instances applied on incisions that are made on the skin to treat particular ailments.

#### Green leaves

The leaves are crushed, and sometimes soaked in water and the resultant concoction may be drunk, or applied directly on the affected area such as in the treatment of toothache. The latex may also be applied on the affected area of the skin, an example being in the treatment of allergy.

#### Others

Higher parasites [32], “*Sagorket*” and fruit bodies of fungi “*Lobchon*” that grow on trees (Sporocarps) are also used.

### Plant use

#### Emetics

There are several plants that are used as emetics. The fruits of the bitter gourd, *Cucumis dipsaceus are crushed and administered to the patient to induce vomiting. The fruits of Gardenia spp*. are also crushed and given to the patient to induce both vomiting and diarrhea. The concoction is considered to be dangerous on overdose, and is therefore only administered by an experienced herbalist. Others used include the leaves of *Calotropis procera* and roots of *Vernonia brachycalyx* which are boiled and the decoctions administered.

#### Purgatives

The roots of *Croton megalocarpus* are boiled and the decoction used as a purgative. Others include the barks from *Pavetta abyssinica* and *Ekebergia rueppelliana*.

#### Heart burn

The treatment involves licking of the ashes prepared from the leaves and twigs of *Cirsium vulgare*. An alternative treatment involves the administration of decoction from the boiled roots of *Croton ciliatoglandulifer*. The leaves of *Dactyloctenium aegyptium* are also crushed and administered to patient. Similarly, the leaves of *Capparis cartilaginea* are chewed for the relief of both heartburn and treatment of peptic ulcers. We interviewed two patients who informed us that upon chewing the leaves, they felt as though “smoke came out of their nose”, before getting some relief. The ash of *Bryophyta* spp. and that obtained from burning the tip end of the fruit of *Ensete ventricosum* are also licked.

#### Stomachache

The tuber of *Cyperus esculentus* is chewed and swallowed. The roots of *Indigoferra arrecta* and bark of *Ehretia cymose var. silvatica* are boiled and decoction taken to relieve stomachache. Alternative cure involves chewing of the leaves of *Warburgia ugandensis*. The roots of *Saba comorensis* and the bark of *Sclerocarya birrea* are boiled and administered to the patient. In the treatment of the stomachache resulting food poisoning, the roots of *Sphaeranthus ukambensis* are chewed or boiled.

#### Abdominal pains (colic pains)

Treatment involves the boiling of the bark of *Acacia hockii* and the roots of *Nuxia congesta*. The tuber of *Cyperus esculentus* and bark of *Acacia nilotica* are also chewed to relieve the colic pain. Alternative treatment involves the chewing of the roots of *Solanum incanum and Croton ciliatoglandulifer*. The barks of *Ziziphus mauritiana var spina Christi* and *Terminalia brownie* may either be chewed or boiled to treat the condition. The leaves of *Justica spp*. may also be used.

#### Colic pain in children

The roots and leaves of *Hoslundia opposite* or tuber of *Cyperus esculentus* are crushed or chewed and administered to the children. An alternative treatment involves the administration of chewed leaves or decoction from boiled roots of *Solanum incanum*.

#### Amoebiasis

A concoction made from the boiled roots and leaves of both *Hoslundia opposite* and *Withania somnifera* leaves of *Lippia javanica* and the bark and seeds of *Zanthoxylun chalybeum*.

#### Kipei condition

This condition manifests with oral thrush and severe abdominal pain. It is treated by using the ashes made from higher parasites [32], “*Sagorket*” that grow on *Croton dichogamus* which are licked. The leaves of *Clerodendrum myricoides* and “*Turesio*” are also burnt and ashes licked.

#### Period pains

The bark of *Faurea saligna* is boiled and concoction administered.

#### Headache

The bark of *Warburgia ugandensis* is burnt and smoke sniffed.

#### Toothache

The leaves of *Fuerstia africana* and/or roots of *Indigoferra arrecta* are crushed and applied on the affected tooth. The leaves of *Warburgia ugandensis* may also be used. An alternative treatment involves the application of the whole the crushed *Spilanthes mauritiana* plant on the site.

#### Teething in children

The ashes of the *Bryophyta* spp.are rubbed on the gums to relive the pain and soreness associated with teething.

#### Malaria and fever

The bark of *Terminalia spinosa* alone, or in combination with that of *Zanthoxylun chalybeum* are boiled and the concoction administered. The fruits and bark of *Pentas longiflora* may also be added. To reduce the fever associated with malaria especially in young children, the bark *Diospyros scabra* is boiled and the patient is bathed in the concoction.

#### Jaundice

Treatment involves the chewing of the bark of *Terminalia brownii* or administration of the decoction obtained by the boiling of the bark of the same plant.

#### Common colds and cough

The decoction from boiled roots of *Uvaria sp var. scheffleri* plant is combined with leaves of either *Zehneria scabra* or *Warburgia ugandensis* which are chewed for treatment of colds including sore throats. The boiled roots of *Nuxia congesta* are used in the treatment of flu. The dried resin (“*Manga*”) of *Schefflera volkensii* is inhaled to clear blocked nose.

#### Chest congestion (wheezing)

The whole of *Caralluma acutangula* is crushed and administered, especially children. An alternative treatment involves use of a concoction from the roots, bark and flowers of *Croton dichogamus* are boiled and administered.

#### Oral thrush

The Whole of crushed *Spilanthes mauritiana* is chewed. For oral thrush in children, they are crushed and mixed with water or milk and administered. Alternative treatment involves gargling of the crushed leaves of *Warburgia ugandensis* or *Hoslundia opposite*.

#### Eye ailments

The leaves of *Ocimum basilicum* are crushed and concoction applied to the eye. The leaves *Fuerstia Africana* of are also crushed and instilled on the affected eye. An alternative treatment involves the grinding the dried barks of either *Olea Africana* and applying the powder into the affected eye. The powder from *Podocarpus gracilior* may also be used.

#### Wounds

The seeds of *Ximenia Americana* are crushed and the oil extracted applied on the wounds. Alternatively, the leaves of *Aloe spp*. are broken and the sap squeezed on to the exposed wounds. The leaves of either *Capparis cartilaginea* or *Indigofera arrecta* are used in the treatment of skin sores and ulcers; whereas those of *Withania somnifera* are used in treatment of chronic skin ulcers. *Aloe spp*. was reportedly used for the treatment of wounds in Nandi and ulcers in Sabaot [20,21].

#### Cancer

Ashes from burnt fruit bodies of fungi “*Lobchon*” that grow on trees (Sporocarps) such as *Acacia spp*., *Faidherbia albida*, *Combretum apiculatum*, *Albizia species* and *Pappea capensis* are mixed with milk and administered to the patient. The roots of *Indigofera arrecta* may also be boiled or chewed and combined with the herbal concoction. *Drimia indica* and *Albuca bracteata* are also used in the treatment of cancer and may be combined with the other herbs. An alternative treatment involves the use of *Zehneria scabra*. A mixture of the whole plant, together with the bark and roots of “*Turesio*”, one maize cob and bean husks are burnt. The resultant ashes are then mixed with ground finger millet and administered. The whole crushed plant of *Portulaca oleracea* may also be used. Researchers from previous studies have reported cytotoxic effects from the isolates of *Acacia spp*. [33], *Albizia species*[34] and *Zehneria scabra*[35].

#### Skin rashes

The crushed leaves of *Commicarpus africanus* are combined with those of *Acacia brevispica* and rubbed on affected area. The bark of *Olea Africana* may also be boiled and the decoction taken to treat the itchy rashes.

#### Removal of ganglions

The leaves of *Acacia brevispica* are crushed and mixed with those of *Commicarpus africanus* and rubbed on the area of the body with a ganglion cyst. It is repeated until the ganglion disappears. We interviewed one patient who informed us that the ganglion disappeared completely after applying the preparation for three days.

#### Herbs used during craniotomy

The whole of *Spilanthes mauritiana* is crushed and soaked in water. It is then administered to patient on the eve of surgery, and for the next two days after surgery. The sap of *Aloe sp*. is then applied on the wound to reduce infection. In order to enhance recovery, the bark of *Zanthoxylun chalybeum* and the whole of *Zehneria scabra* plant are crushed and the resultant concoction administered to the patient.

#### Sexually transmitted diseases

*Acacia melifera* is used as an antibiotic for the treatment of venereal diseases. The roots are boiled and the decoction administered to the patient. It is combined with the whole of *Ipomoea lapidosa* plant and roots of *Berchemia discolor*.

#### Hypertension

The bark of *Prunus Africana* mixed with that of *Podocarpus graciliar* are boiled and administered to the patient in the treatment of hypertension. The concoction made from the tuber of *Rumex acetosella* may also be used.

#### Diabetes

The tubers of *Rumex acetosella* are boiled alone, or in combination with the bark of *Sclerocarya birrea* and the decoction administered.

#### Obesity

The seeds of *Boscia coriacea* are boiled and administered to the patient.

#### Arthritis

The tubers of *Ornithogalum tenuifolium* are crushed and applied on the affected knees or joints. The roots of *Crassocephalum mannii* are boiled and administered.

#### Rheumatism

Higher plant parasites that grow on the following trees; *Acacia mellifera*, *Zanthoxylun chalybeum* and *Pappea capensis* are burnt and the ashes licked.

#### Poultice

The leaves of *Kalanchoe germanae* are pound and rubbed on the painful areas of the body. In the Sabaot study, *Kalanchoe mitejea is used*[21].

#### Erectile dysfunction

The roots of *Berchemia discolor* mixed with those of *Clutia abyssinica* are boiled and the concoction drunk to improve virility. An alternative treatment involves boiling the concoction from the bark and chewing the roasted seeds of *Sterculia aficana*. Young shoots of the whole plant of *Tribulus terrestris* may also be chewed.

#### Renal disorders/enlarged prostate

The fruits of *Saba comorensis* are used in the diagnosis of renal disorders including an enlarged prostate. It is also used in the diagnosis of sexual function, to ascertain whether the man is responsible in families who cannot bear children. The herbalists informed us that it was difficult to obtain the fruits as the plant has yellow sweet fruits which are a delicacy for baboons. The plant is used only in men, and appears to have some diuretic activity. In order to make a diagnosis of a renal disorder, the patient is given the fruits, and if the patient does not pass yellow urine after about one hour, then it is assumed that he has a renal disorder especially an enlarged prostate, or a venereal disease. It is also interpreted to mean that he has erectile dysfunction and therefore sterile, if they have been in a marriage and have not been able to get children. A concoction made from the bark of *Prunus africana* is also used on the treatment of an enlarged prostate.

#### Mumps

The barks of Erythrina abyssinica are used in the treatment of mumps. They are boiled and concoction administered.

#### Lactation

In order to enhance lactation in breastfeeding mothers, the leaves of *Dolichos spp* are used. They are dried, crushed and dissolved in water before administering to the mother. An alternative preparation consists of the use of crushed leaves and twigs of *Lantana trifolia*.

#### Abortifacients

The bark of *Albizia spp* is boiled and administered to induce abortion. It is also used as a contraceptive. The leaves and seeds of *Cassia occidentalis*/*obtusifolia* and stem of *Cyphostemma serpens* are also used as abortifacients. They are crushed and soaked in water before administration.

#### Removal of placenta

In case of a retained placenta in both humans and animals, the bark of *Heeria reticulata* is crushed, dissolved in water and administered.

#### Antihaemorrhagic

The bark of *Cassia didymobotrya* is used to stop bleeding. The dried bark is ground and powder applied on the bleeding area.

#### Anaemia

Iron defiency anaemia is treated by supplementing food with *Crotalaria brevidens*.

#### Heavy snoring

The leaves of *Coccinia grandis* are used as a remedy to treat people who snore heavily. Patients are advised to use the leaves as vegetables.

#### Astringent

The leaves and twigs of *Lantana trifolia* are used as astringents.

#### General malaise/appetizers

The roots of *Carissa edulis* are boiled and decoction administered to the patient with general malaise symptoms. Ripe fruits of the same plant are used as appetizers. The bark of “*Seremwo*” is also boiled and administered to patient for the same purpose.

#### Natural healing vegetables and fruits

There are some specific vegetables that are recommended for use by convalescing patients or those in frail health. After a course of treatment, the spider plant *Gynandropsis gynandra* is cooked and milk added. It is then administered to the patients in order to speed up the recovery. Other healing vegetables include the leaves of *Digera muricata*, *Crotalaria incana* and *Leptadenia hastate*. It also includes the tender young plant of *Tribulus terrestris*, the whole of *Dactyloctenium aegyptium* and *Portulca qudrifida* plants, as well as the leaves and oil from the seeds of *Balanites aegyptiaca*. The fruits of *Vangueria madagascariensis* and *Vangueria apiculata* are used by recuperating patients.

#### Food flavours

The leaves of *Lippia javanica* are used. There are three species of this plant used, but only one is used for this purpose and is identifiable by experienced herbalists. The plant is a potent sweetener and is boiled with food, especially maize in order to improve the taste. The flowers of *Capparis cartilaginea* are also used, especially in the preparation of fish stew to reduce the smell as fish is not generally popular among the Kalenjin community.

#### Herbal “tea”

The barks of *Ziziphus Mauritania var. spina Christi* and *Acacia hockii* are boiled in water used as herbal beverages in a similar way as tea leaves. The bark of *Psiadia Arabica* may also be used.

#### Scorpion, bee, and wasp stings

The leaves of *Acalypha fruticosa* are applied directly on the stung area of the body.

#### Antivenom

The bark of *Euclea divinorum* and roots of *Tragia brevipes* are used as antivenom. They are crushed and the resultant preparation applied into an incision made on the area that has been bitten by the snake. The juice from the fruits of *Solanum incanum* combined with the crushed bark of *Lannea fulva* are then applied on the bitten area. The sap from the leaves of *Sansevieria intermedia* are used to kill snakes. The leaves are squeezed and juice applied on the opening of the hole where snakes reside. The authors in the Sabaot study also reported the use of *Euclea divinorum* for the treatment of snake bites [21].

#### Talisman

It is a common practice to make three incisions on the temples, nape and epigastric region among the Marakwet and Pokot tribes in children or a person who has been ill for a long time in order to protect them from witches or people with “bad eyes” (evil intentions) or evil influences [13]. The burnt ashes of *Cirsium vulgare* are used for this purpose. The tuber of *Cyperus esculentus* is also chewed directly and the saliva smeared on children where it acts as a talisman.

#### Drug of abuse

“*Mindililwo ne mining*”, is an ephemeral plant that is usually chewed to provide a sweet taste, but the elders informed us that they know it as a drug of abuse when the leaves are smoked. In order to discourage children from going near the plant; they are informed that the plant only grows near where “*Ilat*” lives; and that it is “*nguekab chesowiloy*” (a vegetable that belongs to the devil). “*Ilat*” is considered an agent of “*Asis*”, the Supreme Being, and omnipotent arbiter of all things and guarantor of right. “*Ilat*”, in mundane terms “thunder and lightning” is dreaded, for he can cause death. He is invincible, and is seen when lightning strikes something, such as a house or a tree. He acts in the interest of justice and may strike an offenders house or stock [13].

#### Ceremonial

Among the Marakwet, and indeed all Kalenjin communities, *Periploca linearifolia* is considered a sacred plant. It is used in all celebration ceremonies including weddings and initiations [20,21,36].

#### Perfumes

The leaves of *Iboza spp*. are crushed and applied to the body as perfume. The dried resin obtained from *Schefflera volkensii* is added to tobacco to improve the aroma and can also be applied on the body.

#### Fermentation

The fruits of *Kigelia Africana* and *Flacourtia indica*, and the leaves of *Grewia villosa* and *Croton dichogamus* are used to provide fermentation during the brewing of traditional beer.

#### Hides and skins

The seeds of *Ximenia americana* and *castor plant* are used for the treatment of hides and skins.

#### Toothbrush

The twigs of the following tree species are used as toothbrushes: *Salvadora persica*, *Dodonaea viscosa*, *Diospyros spp*., *Cordia sinensis*, *Euclea divinorum* and *Grewia bicolor*.

#### Arrow poison

*Acokanthera schimperi* is used as an arrow poison. The roots are boiled and left to settle and after evaporation, the concentrate applied on the arrows. The efficacy of the poison is then tested in dogs. In order to enhance the potency, the poison is mixed with bile from either a crocodile or monitor lizard. The plant has been used as an arrow poison in many parts of Africa [37-39]. However, in the Nandi study *Acokanthera schimperi* was reportedly used as an antibiotic for the treatment of syphilis [20].

#### Pesticides

The tuber of *Cyphostemma cyphopetalum* is used to eradicate all insects. It is crushed water added, and the resultant concoction is used against all insects. When used to kill household insects like mites, the concoction is left standing in the room for one day and after that left for at least two before occupation since the plant is very poisonous. It is also employed as a poison to kill unwanted animals. The leaves and fruits of *Periploca linearifolia* are used for the eradication of lice in goats and sheep. The whole plant is crushed and soaked in water and applied on the skin of the animals. It is also used as a poison. The fruits of *Balanites rotundifolia* are also employed as pesticides. They are crushed, soaked in water and used against insect pests in both crops and animals. The leaves and twigs of *Hypoestes forskaolii* are crushed and used to kill pests especially lice on domestic animals.

### Veterinary uses

#### Poisoning/constipation

The whole of *Maesa lanceolata* plant is crushed and after the addition of a little water administered to an animal which has eaten poison or constipated to induce diarrhoea. Alternatively, the leaves of *Heeria reticulata* are crushed and administered.

Our biggest challenge was to gain the trust from the herbalists. It took a lot of persuasion and involvement of opinion leaders to let them understand our intentions and volunteer the information. Some conditions described by the herbalists were not easily identifiable as we could not access the patients, or they were already healed by the time we met them. Herbalists from the same area tended to use the similar herbs for the same conditions. However, we found many incidences of herbalists from a similar location using the same plants for totally different conditions, especially those widely known to specialize in the treatment of a specific disorder such as infertility. This could be attributed to the fact that the traditional knowledge is by and large a closely guarded family secret [7].

Emetics and purgatives were widely used. Among the Kalenjin community, emesis “*Ketap*” and diarrhoea “*Kegoor*” constituted important forms of treatment as they were believed to decontaminate the body from pollutants, just like their Maasai counterparts [40,41]. It was therefore important for each person to use either purgatives, emetics or in most instances both at least once a year in order to cleanse the system so as to maintain good health; or to prevent a disease condition that they referred to as “*N*’*gwono*”. The term “*N*’*gwono*” is also separately a synonym for poison. “*N*’*gwono*” was a very prominent condition that was mentioned by the herbalists. They described the condition as presenting with malaria like symptoms including malaise, fatigue, lack of appetite, nausea and vomiting, including the vomiting of a yellow substance; presumably bile. The condition according to the herbalists could only be treated by inducing vomiting and diarrhoea by use of emetics and purgatives. Both classes of herbal drugs were also taken in preparation of major events such as festive seasons. In fact, emesis and diarrhoea were mandatory for boys or girls as part of the preparations, weeks before undergoing circumcision. However, the herbalists informed us that some of the emetics and purgatives such as *Gardenia Spp*. were quite toxic on overdose and could only be administered by experienced herbalists. *Calotropis procera* in large doses is known to be an emetic and a purgative [42,43]. *Croton megalocarpus* has also been used as a purgative and *Pavetta abyssinica* for indigestion [18].

Our research team could not quite figure out the condition that most of the herbalists and patients we interviewed referred to as cancer, “*Seryan*”. The symptoms they described appeared to suggest myasis, chronic tropical ulcer, or probably squamous cell carcinoma. They described cancer as a condition that manifests with a chronic wound on the skin. After treatment using the cancer specific herbs for some period of time, the area of the skin with the ulcer reduced, finally into a small size which they described as the mouth, “*Kuti*”. Upon pressing the areas around the “mouth”, a substance resembling a pupa emerging from a cocoon which they referred to as “*Kutyan*” appears, indicating that the patient was now healed. This could also suggest guinea worm infestation although unlikely as this is not a Guinea worm endemic area. We concluded that this was most likely thick solid pus from an abscess, suggesting involvement of some infection. Had we seen a patient with the condition before treatment, then perhaps we would have made a diagnosis. However, the fact that some of the plants used in the treatment of the condition such as *Acacia spp*., *Albizia species* and *Zehneria scabra* have been shown to possess some cytotoxic activities may actually suggest that it is actually cancer [33-35]. Indeed cancer was the condition treated by the highest number of species, indicating the complexity of the condition (Table 5). “*Kipei*” was another condition that was not very clear. The herbalists described the condition as that presenting with severe abdominal pain and oral thrush, suggestive of some form of immunosuppression. Likewise, we were not able to see a patient with this condition. However, we managed to interview some other patients with other conditions, such as ulcers and ganglion growths.

Despite the long distance between Marakwet and Sabaot districts (about 200 kilometers away), there were 24 recorded plant species that were used in both communities, with 9 of them having similar uses. These include: *Acacia lahai*, *Albezia spp*., *Aloe spp*., *Carissa edulis*, *Clerodendrum myricoides*, *Diospyros spp*., *Dolichos spp*., *Ensete ventricosum*, *Erythrina abyssinica*, *Euclea divinorum*, *Flacourtia indica*, *Indigofera arrecta*, *Kalanchoe germanae*, *Lantana trifolia*, *Maesa lanceolata*, *Periploca linearifolia*, *Prunus Africana*, *Ricinus communis*, *Schefflera volkensii*, *Cassia* (*Senna*) *didymobotrya*, *Solanum incanum*, *Vangueria apiculata*, *Warburgia ugandensis*, *Withania somnifera*[21]. The nine plants used for similar indications were: *Aloe spp*. (wounds/ulcers), *Dolichos spp*. (enhance lactation/fertility), *Euclea divinorum* (antivenom), *Kalanchoe spp*. (*poultice*), *Periploca linearifolia* (ceremonial/initiation), *Prunus Africana* (UTIs), *Schefflera volkensii *(respiratory disorders), *Solanum incanum *(abdominal pains) and *Warburgia ugandensis* (headache).

The species that were common with Nandi were (eight) including: *Acokanthera schimperi*, *Aloe spp*., *Carissa edulis*, *Justica spp*., *Cassia* (*Senna*) *didymobotrya*, *Ehretia cymose*, *Justica spp*., *Kigelia Africana*, *Periploca linearifolia*, with four having similar indications [20]. The 4 plants with similar indications were: *Aloe spp*.(wounds), *Ehretia cymose* (stomachache/typhoid), *Justica spp*.(abdominal pains/ulcers), *Periploca linearifolia* (ceremonial/rituals). Four plants; *Aloe spp*., *Carissa edulis*, *Cassia* (*Senna*) *didymobotrya*, and *Periploca linearifolia* were commonly used in all the three districts; with *Aloe spp*. and *Periploca linearifolia* having similar uses in all the three districts.

## Conclusion

The study provides a comprehensive report on the vast wealth of traditional medical knowledge, health practices and plant use among the Marakwet community. Scientific evaluation of the medicinal plants may lead to the development of new drugs. There are few records on traditional medicinal plant usage among the various communities in Kenya despite their widespread use. There is therefore urgent need to document this information, as it is rapidly disappearing due to influence of western medicine and other reasons including socio-cultural issues and overexploitation coupled with rapid deforestation. It is important to collect this information and develop a database of medicinal plants for future research and potential development of new drugs.

## Competing interests

The author’s declare that they have no competing interests.

## Authors’ contributions

All the authors shared the contributions to the work fieldwork of the manuscript. Kipkore and Wanjohi identified the plants. Kigen and Rono interviewed the herbalists and patients, in order to identify the illnesses. Kipkore and Kigen analyzed the data and prepared the manuscript. All authors read and approved the final manuscript.
